# Association between obstructive sleep apnea and risk of lung cancer: findings from a collection of cohort studies and Mendelian randomization analysis

**DOI:** 10.3389/fonc.2024.1346809

**Published:** 2024-07-12

**Authors:** Jun Yao, Ran Duan, Qingyuan Li, Ruonan Mo, Pengcheng Zheng, Tong Feng

**Affiliations:** ^1^ Respiratory and Critical Care Department, Guangyuan Central Hospital, Guangyuan, Sichuan, China; ^2^ Clinical Medical College, Chengdu Medical College, Chengdu, Sichuan, China; ^3^ Department of Oncology, Clinical Medical College and The First Affiliated Hospital of Chengdu Medical College, Chengdu, China; ^4^ Respiratory and Critical Care Department, The First Affiliated Hospital of Chengdu Medical College, Chengdu, Sichuan, China; ^5^ The Second School of Clinical Medicine, Southern Medical University, Guangzhou, China

**Keywords:** lung cancer risk, obstructive sleep apnea, meta-analysis, Mendelian randomization, cohort studies

## Abstract

**Background:**

Previous cohort studies conducted on large populations have suggested a potential association between obstructive sleep apnea (OSA) and an elevated risk of developing lung cancer. However, limited research has comprehensively investigated the correlation between the two conditions, and the causal effect remains unknown.

**Methods:**

A comprehensive and systematic search was conducted across various databases, including PubMed, Web of Science, Cochrane Library, and Embase, from their inception dates to November 1, 2023. To assess the relationship between OSA and lung cancer, a meta-analysis was performed. Additionally, a two-sample Mendelian randomization (MR) study was conducted using summary data. The datasets included 336,659 individuals from the FinnGen study for OSA and 27,209 individuals from the International Lung Cancer Consortium study, as well as 420,473 individuals from the UK Biobank study for lung cancer. The estimates from each study were aggregated using the inverse variance-weighted method.

**Results:**

Data from six population-based cohort studies, encompassing 6,589,725 individuals, indicated a significant increase in the risk of developing lung cancer among patients with OSA (HR 1.28, 95% CI 1.07-1.54). However, the MR analysis did not support a causal relationship between OSA and lung cancer (OR 1.001, 95% CI 0.929–1.100). This lack of association was consistent across specific subtypes of lung cancer, including non-small-cell lung cancer (OR 1.000, 95% CI 0.999–1.000, p = 0.974), lung adenocarcinoma (OR 0.996, 95% CI 0.906–1.094, p = 0.927), and squamous cell lung carcinoma (OR 1.034, 95% CI 0.937–1.140, p = 0.507).

**Conclusions:**

Our meta-analysis findings suggest an elevated risk of lung cancer among individuals with OSA. However, the MR analysis did not provide evidence supporting a causal relationship between OSA and lung cancer. Further investigation is required to uncover the underlying factors contributing to the observed association between OSA and lung cancer risk.

## 1 Introduction

Cancer remains a pressing global health concern and is responsible for a staggering number of deaths on a worldwide scale. In particular, lung cancer emerges as the foremost culprit, being the leading cause of cancer-related fatalities and contributing to a staggering 1.8 million lives lost. Recent data from the year 2020 delineates that lung cancer constituted a significant proportion of newly diagnosed cases, accounting for 11.4% of all cancer diagnoses (1). Obstructive sleep apnea (OSA), a condition characterized by interrupted breathing during sleep, affects a staggering number of adults globally, with estimates suggesting that nearly one billion adults suffer from this sleep disorder (2). Growing evidence indicates that OSA plays a significant role for various types of lung cancer. The underlying mechanisms involve intermittent hypoxia, oxidative stress, and inflammation, all of which contribute to the development and progression of cancer within the body (3). Various observational studies have provided evidence indicating a greater incidence of lung cancer in individuals with OSA (4, 5), and patients diagnosed with lung cancer also exhibit a higher incidence of OSA (6, 7). Nevertheless, conflicting evidence from other observational studies undermines the establishment of this association (8, 9). Moreover, it is vital to acknowledge and carefully consider the limitations present in prior research, which encompass factors such as small sample sizes, dependence on local registries, and ambiguous diagnostic criteria. These limitations have the potential to impact the findings.

The main objective of our study was to conduct a comprehensive meta-analysis of existing population-based cohort studies in order to thoroughly examine the relationship between OSA and lung cancer. It is imperative to recognize that observational studies possess inherent limitations, as they solely establish correlations without determining causation between OSA and lung cancer. This limitation arises from the potential impact of confounding variables or reverse causation. Additionally, while meta-analysis yields valuable insights, it does not definitively establish a causal relationship between OSA and lung cancer. Since most studies rely on observational data, there is a potential for reverse causation bias, where the association may not be due to OSA itself but rather the lung cancer causing OSA. Traditional observational studies may have limitations, including the presence of confounding biases. OSA and lung cancer share common risk factors and comorbidities, such as obesity, male gender, advanced age, smoking, and chronic obstructive pulmonary disease (COPD) (10, 11). These shared factors can complicate efforts to establish a causal relationship between OSA and lung cancer, making it challenging to accurately determine the impact of OSA on the development of specific lung cancers. Therefore, a more comprehensive understanding of the causal association between OSA and lung cancer is crucial for preventing potential adverse outcomes.

Mendelian randomization (MR) is a widely employed technique for inferring credible causal relationships in cases where conducting randomized controlled trials (RCTs) is impracticable (12). By capitalizing on the genetic variations that occur during meiosis, independent of any environmental or acquired factors, the MR design offers a valuable tool for randomization. This mechanism helps reduce the impact of any remaining confounding variables and potential reverse causality, making it an ideal approach for minimizing interference in studies (13). Recent MR studies have successfully identified causal associations between body mass index (BMI) and the development of lung cancer (14, 15). However, a dearth of MR evidence currently exists to support a causal relationship between OSA and lung cancer.

Given this knowledge gap, our study aims to address this lacuna by conducting an two-sample MR analyses on two large databases, aiming to achieve a sufficient sample size, which had been typically constrained due to the low occurrence of lung cancer in previous observational studies. Through examining the causal effect of OSA on the lung cancer, we seek to provide compelling evidence for its role in the genesis of this disease. Such findings would offer a novel perspective on the early detection of lung cancer, ultimately enhancing patient outcomes.

## 2 Methods

The current investigation followed the protocols and recommendations outlined in Cochrane’s Handbook for its research methodology. Report list for strengthening meta analysis can be found in **Supplementary Table 1**. To ensure transparency and credibility, the registration of our study has been completed and recorded, with a unique identifier assigned as PROSPERO ID 480577. Detailed methodology is provided in the **Supplementary Materials**.

### 2.1 Meta analysis

We conducted comprehensive searches on PubMed, Web of Science, Cochrane Library, and Embase on November 1, 2023, using terms related to OSA and various cancers, and reviewed references of relevant articles. Included studies were longitudinal follow-up studies with OSA patients, assessing OSA and lung cancer incidence over at least 3 years, and reporting hazard ratios (HR) adjusted for confounders. We excluded non-cohort studies, those only reporting lung cancer mortality, case reports, conference papers, reviews, animal studies, and non-English studies. Two reviewers independently extracted data on author, publication year, participant characteristics, OSA assessment methods, follow-up duration, lung cancer validation, and covariates. We used the Newcastle-Ottawa Scale (NOS) to assess study quality, categorizing them into high, medium, and low quality. We synthesized HRs and 95% confidence intervals (CI) using meta-analysis, assessed heterogeneity with Cochran’s Q test and the I² statistic, and used random-effects or fixed-effects models accordingly. Sensitivity analyses included studies accounting for smoking status, and pre-specified subgroup analyses were based on follow-up duration. Limited study numbers prevented in-depth subgroup analyses or funnel plot asymmetry assessments. Statistical analyses were performed using RevMan software (Version 5.3; Cochrane Collaboration, Oxford, UK). Detailed methodology is provided in the **Supplementary Materials**.

### 2.2 Study design of MR analysis

**Figure 1:**
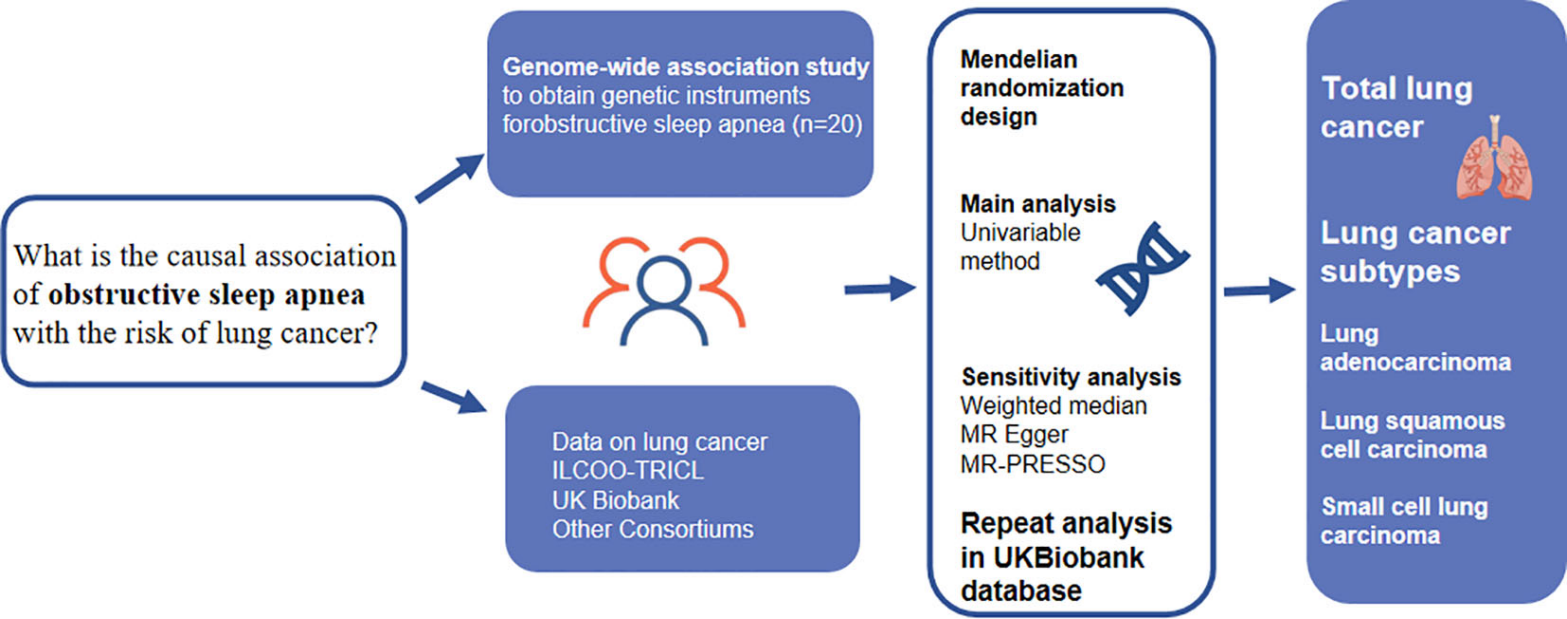
Overview of the design and main findings from a Mendelian randomization (MR) study. MR, Mendelian randomization. MR-PRESSO, MR pleiotropy residual sum and outlier test; IVW, inverse-variance weighted.

**Figure 1** presents a comprehensive overview of the study design. To comprehensively understand the causal effects of OSA on total lung cancer and its various histological types, we utilized the most extensive available dataset on OSA as well as two extensive lung cancer datasets.To ensure the validity of our results, we relied on three key assumptions concerning the genetic variants used in our analysis (16). First, these variants were selected based on their reliable and robust association with the exposure under investigation. Second, we assumed that they were independent of any factors that could potentially confound the relationship between the exposure and the outcome. Lastly, we assumed that these variants solely influenced the outcome through their impact on the exposure variable. All the genome-wide association study (GWAS) summary statistics employed in our study were openly accessible, and we sought ethical approval from the original studies.

### 2.3 Data sources and instrument variables

#### 2.3.1 Obstructive sleep apnea

In this study, we meticulously selected a total of twenty single nucleotide polymorphisms (SNPs) that have recently demonstrated a significant association with traits relevant to OSA in individuals of European descent. The cohort for this GWAS comprised 336,659 individuals of European ancestry (17). The data for OSA patients were obtained from nationwide health registries in Finland, with a total of 38,998 patients identified as having OSA based on the diagnosis criteria. The diagnosis involved considering OSA-related symptoms, conducting clinical examinations, and analyzing national insurance data using the apnea-hypopnea index (AHI), with thresholds set at a minimum of 5 events per hour (17). Further information regarding the selected SNPs as instrumental variables can be found in **Supplementary Table 4**.

#### 2.3.2 Lung cancer

The data for lung cancer obtained in this study came from two reliable and extensive databases - the International Lung Cancer Consortium (ILCCO) and the UK Biobank. The ILCCO is a collaborative project focused on investigating the genetic variations associated with lung cancer (18). On the other hand, the UK Biobank is a comprehensive research endeavor that includes a large population-based cohort of over 500,000 individuals from diverse regions across Great Britain, providing valuable biological samples (19). For our analysis, we utilized two sets of GWAS summary statistics from ILCCO and the UK Biobank as our primary outcomes. The ILCCO data set comprised 11,348 cases and 15,861 controls, while the UK Biobank data set included 4,030 cases and 238,678 controls. In addition to these primary outcomes, we also examined GWAS statistics for specific subtypes of lung cancer. This includes lung adenocarcinoma (3,442 cases and 14,894 controls), lung squamous cell carcinoma (3,275 cases and 15,038 controls), and small cell lung carcinoma (2,791 cases and 20,580 controls) from the ILCCO database. These secondary outcomes aimed to investigate the association between OSA and the various pathological subtypes of lung cancer. To ensure the reliability of our analysis, we implemented a filter that only included variants with a minimum variant allele frequency greater than 0.01. For a more detailed representation of the data sources for the outcomes, please refer to **Supplementary Table 5**.

#### 2.3.3 Potential pleiotropy

In order to investigate the potential mediating role of OSA on established risk factors for lung cancer, including body mass index (BMI), smoking, and chronic obstructive pulmonary disease (COPD), an analysis was conducted using the inverse variance weighted (IVW) method. The objective was to uncover any mediating effects of OSA on the development of lung cancer, taking into account these known risk factors.To evaluate the connection between OSA and smoking, we utilized genetic instruments obtained from the Sequencing Consortium of Alcohol and Nicotine use (GSCAN) project. This particular project offered a comprehensive genetic analysis of smoking-related traits, such as smoking initiation, smoking duration, and smoking frequency (20). With a dataset of 632,802 individuals of European ancestry, the GSCAN project offers the largest and most reliable source of genetic data for this investigation. In order to investigate the association between OSA and COPD, as well as BMI, summary statistics data were obtained from two reputable sources: the UK Biobank and the Genetic Investigation of Anthropometric Traits (GIANT) (21). These studies are well-established providers of genetic data and include a large sample size of 462,933 individuals for COPD and 681,275 individuals for BMI (**Supplementary Table 5**).

#### 2.3.4 SNP selection

Significant single nucleotide polymorphisms (SNPs) associated with our study outcomes were identified using a threshold criterion of P < 5E-8. These SNPs were found in various gene regions and showed no evidence of linkage disequilibrium, as determined by the 1000 Genomes European reference panel (22). In order to enhance the robustness of our MR design, we excluded SNPs that were strongly linked to the lung cancer (P < 5E-8) to mitigate potential biases. Additionally, we standardized the impact of each SNP on both the outcome and exposure to ensure consistency across alleles (refer to **Supplementary Table 6**). To assess the likelihood of weak instrument bias in the instrumental variables employed, we utilized the F-statistic. This statistical measure was calculated using the equation F = R^2^/(1 - R^2^) * (N - k - 1)/k (23). In this equation, R^2^ represents the proportion of variability in the risk factor that can be attributed to genotype, N represents the sample size, and k represents the number of instrumental variables used (24). An F-statistic value exceeding 10 suggests a low probability of weak instrument bias.

### 2.4 Statistical analyses in MR analysis

#### 2.4.1 Main Mendelian randomization analyses

In our analysis, we employed IVW models as the primary methods of study. These models were individually implemented in each cohort. To aggregate odds ratio (OR) estimates for a specific endpoint from various sources, we adopted a fixed-effects meta-analysis approach. Despite the high accuracy of the IVW method in providing estimates, it does not account for potential biases arising from invalid instruments or pleiotropic effects (25). To ensure the reliability and consistency of our findings, we conducted analyses using both the ILCCO and UK Biobank databases. In the IVW analysis, we utilized the Q statistic and I^2^ index to evaluate the heterogeneity. If there is heterogeneity, we use a random forest model.

#### 2.4.2 Sensitivity analyses

In order to ensure the accuracy and strength of our findings, this investigation employed several sensitivity analyses. The weighted median approach permits a maximum of 50% of instrumental variables to violate the MR assumption in the presence of horizontal pleiotropy (26). In order to identify directional pleiotropy, the intercept derived from MR-Egger regression was utilized (27). To evaluate and rectify horizontal pleiotropy, we utilized the MR-PRESSO technique, which is composed of three components: (a) identification of horizontal pleiotropy, (b) correction by removing outliers, and (c) examination of significant differences in causal estimates before and after outlier correction (28). It is important to emphasize that the MR-PRESSO method is less biased and offers improved precision when compared to both IVW and MR-Egger. Furthermore, a leave-one-out analysis was conducted to assess whether a single SNP was exerting influence or biasing the MR estimate.

All statistical analyses were conducted using the R project version 4.1.3, with the TwoSample MR package utilized for the MR analysis (29).

## 3 Results

### 3.1 Search results and study characteristics

**Figure 2:**
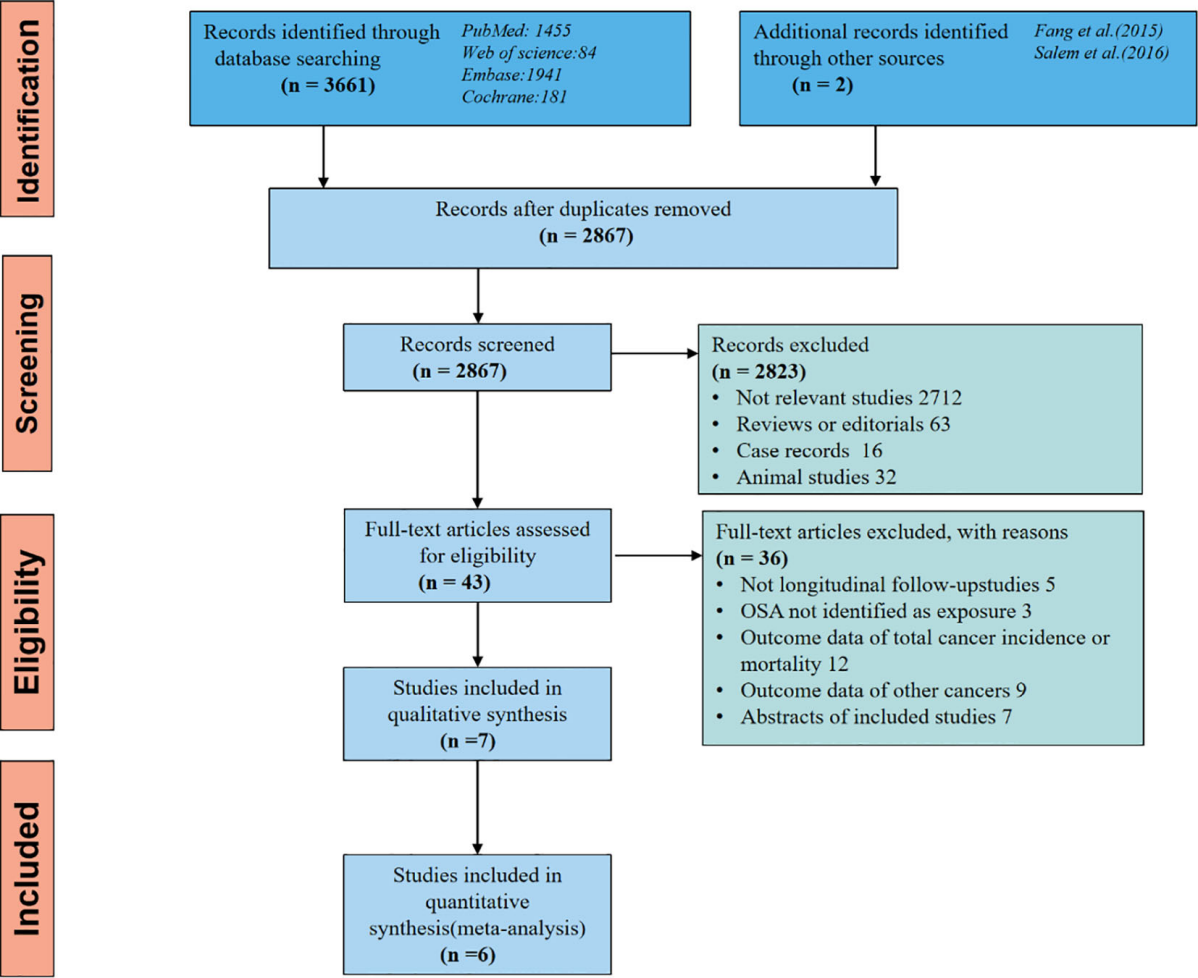
The flow diagram of a detailed overview of the search strategy and the process used for identifying studies included in the meta-analysis.

Initially, our database search yielded 2867 articles, but after eliminating duplicate studies, we were left with 2823 articles that were not relevant to our meta-analysis based on their titles and abstracts. Subsequently, we thoroughly examined the remaining 43 studies through full-text reading. Out of these, we excluded 36 studies for reasons outlined in **Figure 2**, resulting in 7 studies for inclusion in our meta-analysis.

All 7 articles were population-based cohort studies, with 4 originating from America and one each from Korea, Canada, and Australia (5, 8, 9, 30–33).Overall, these studies encompassed a total of 6,589,725 individuals, with 18,879 cases of lung cancer. The duration of follow-up varied between 3 and 11 years across the studies. We present the characteristics of these studies in **Table 1** and Newcastle-Ottawa Scale (NOS) in **Table 2**.

**Table 1:** Characteristics of the included studies in the meta-analysis.

Study	Study Design	Sample Size	Total Incident Lung Cancer	OSA Diagnosis	Lung cancer validation	Setting/ Country	Mean Age	Male (%)	Covariates	Median Follow-Up Duration (Yr)
Gozal 2016 (34)	Retrospective cohort	3,408,906	NR	ICD	ICD	USA	NR	50.2	Age, sex, morbid obesity, hypertension, type 2 diabetes, ischemic heart disease, coronary heart failure, stroke, cardiac arrhythmias, and depression	3,20, 3.75, and 3.91 for different subgroups
Sillah 2018 (33)	Retrospective cohort	34,402	115,	ICD	ICD	USA	51.6	57.4	Age, sex	5.3
Jara 2020 (30)	Retrospective cohort	1,377,285	10,595	ICD	ICD	USA	55.2	94	Age, sex, year of cohort entry, smoking status, alcohol use, obesity, and comorbidity	7.4
Kendzerska 2021 (9)	Retrospective cohort	33,711	241	Polysomnography	Medical chart review	Canada	50	58	Age, study year, clinic site, alcohol use, and comorbidities including hypertension, diabetes mellitus, CVD, and COPD	7
Huang 2021 (5)	Prospective cohort	65,330	492	Self-reported clinical diagnosis of OSA	ICD	USA	73	0	Age, race, family history of cancer, body mass index, height, pack-years of smoking, alcohol drinking, physical activity, sleep duration, duration of hormonal therapy use by type, history of type 2 diabetes, aspirin use, and recent physical examination	8
Marriott 2023 (8)	Retrospective cohort	20,289	173	Polysomnography	ICD	Australian	50	69	age and, in some cases, sex, BMI and smoking status,	11.2
Park 2023 (32)	Retrospective cohort	1,649,802	7263	ICD	ICD	Korea	45.6	75.8	sex, age, subjects’ income levels, diabetes, hypertension, dyslipidemia, stroke, chronic obstructive pulmonary disease, and ischemic heart disease.	5.9

NR, No results.

**Table 2:** Evaluation of risk of bias using the Newcastle-Ottawa Scale (NOS) Cohort NOS.

Study	Representativenes of exposed cohort	Selection of the non-exposed cohort	Ascertainmen t of exposure (secure record, structured interview)	Demonstrate s that cancer was not initially present	Adjusts for age	Adjusts for obesity	Assessment of outcome (record linkage)	Follow -up at least 3 years	Enough long follow-up duration	Total	Risk of bias*
Gozal 2016	1	1	1	1	1	1	1	1	0	8	Low
Huang 2021 (5)	0	1	0	1	0	1	1	1	0	5	Moderate
Jara 2020 (30)	0	1	1	1	1	1	1	1	0	7	Moderate
Kendzersa 2021 (9)	1	1	1	1	1	0	1	1	0	7	Moderate
Sillah 2018 (33)	1	1	1	0	1	0	1	1	0	6	Moderate
Marriott 2023 (8)	1	1	1	1	1	1	1	1	0	8	Low
Park 2023 (32)	1	1	1	1	1	1	1	1	0	8	Low

*high (<5 stars), moderate (5-7 stars), low risk of bias (≥8 stars).

### 3.2 Overall lung cancer risk in OSA patients

**Figure 3:**
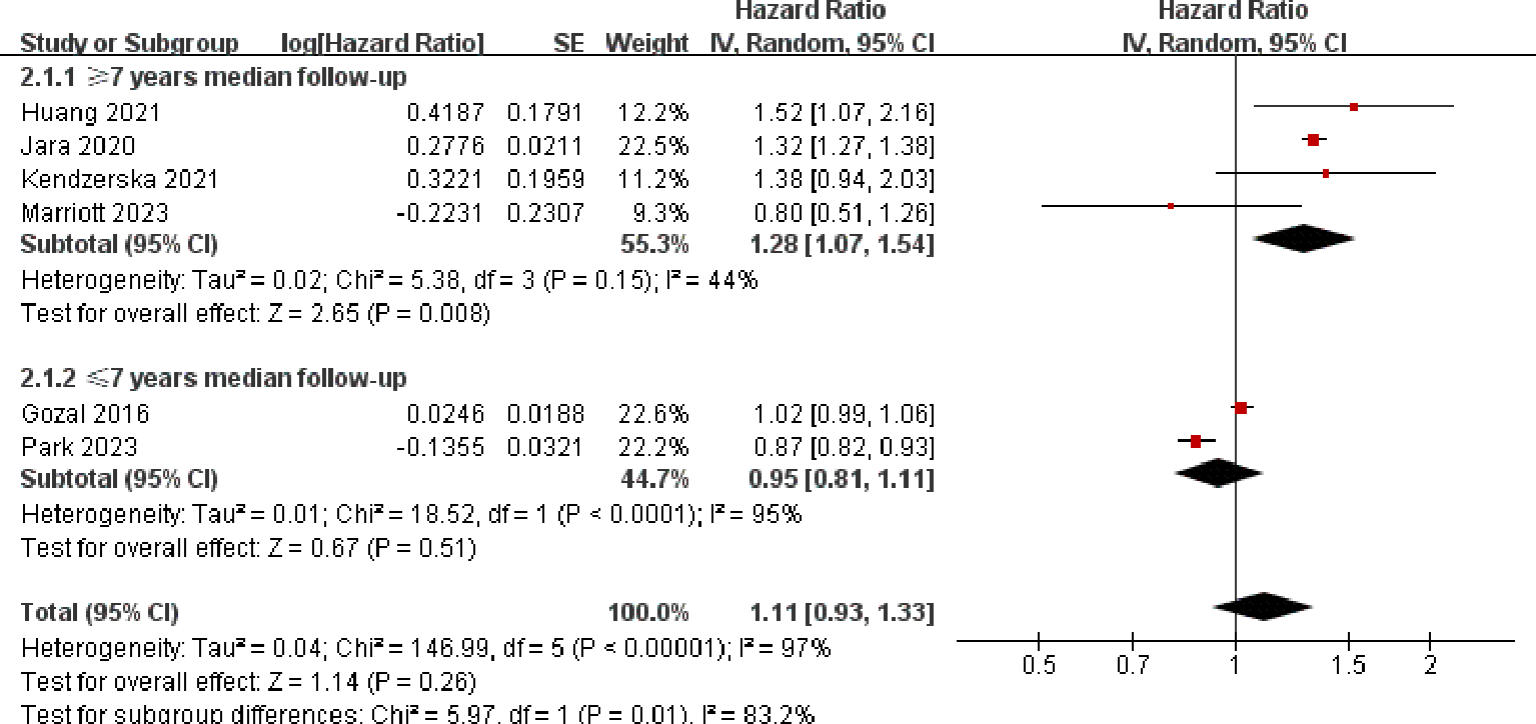
Comparing of Lung Cancer Incidence in Patients With or Without OSA Based on Meta-Analysis.

Regarding the evaluation of quality, all 7 studies incorporated in our meta-analysis indicated a moderate or low potential for bias. However, one particular study conducted by Sillah was not included in the meta-analysis due to inadequate adjustments for age and sex. Moreover, this study did not take into account other coexisting medical conditions, which significantly differed from the rest of the studies.The combined HR for the overall risk of developing lung cancer among individuals with OSA was calculated as 1.11 (95% CI 0.93–1.33). This finding suggests that there is no notable increase in lung cancer risk among individuals with OSA. It is worth noting that the 6 remaining studies exhibited significant heterogeneity (I2 = 97%, p < 0.001), as depicted in **Figure 3** of the comparative analysis.

### 3.3 Subgroup analysis

**Figure 4:**
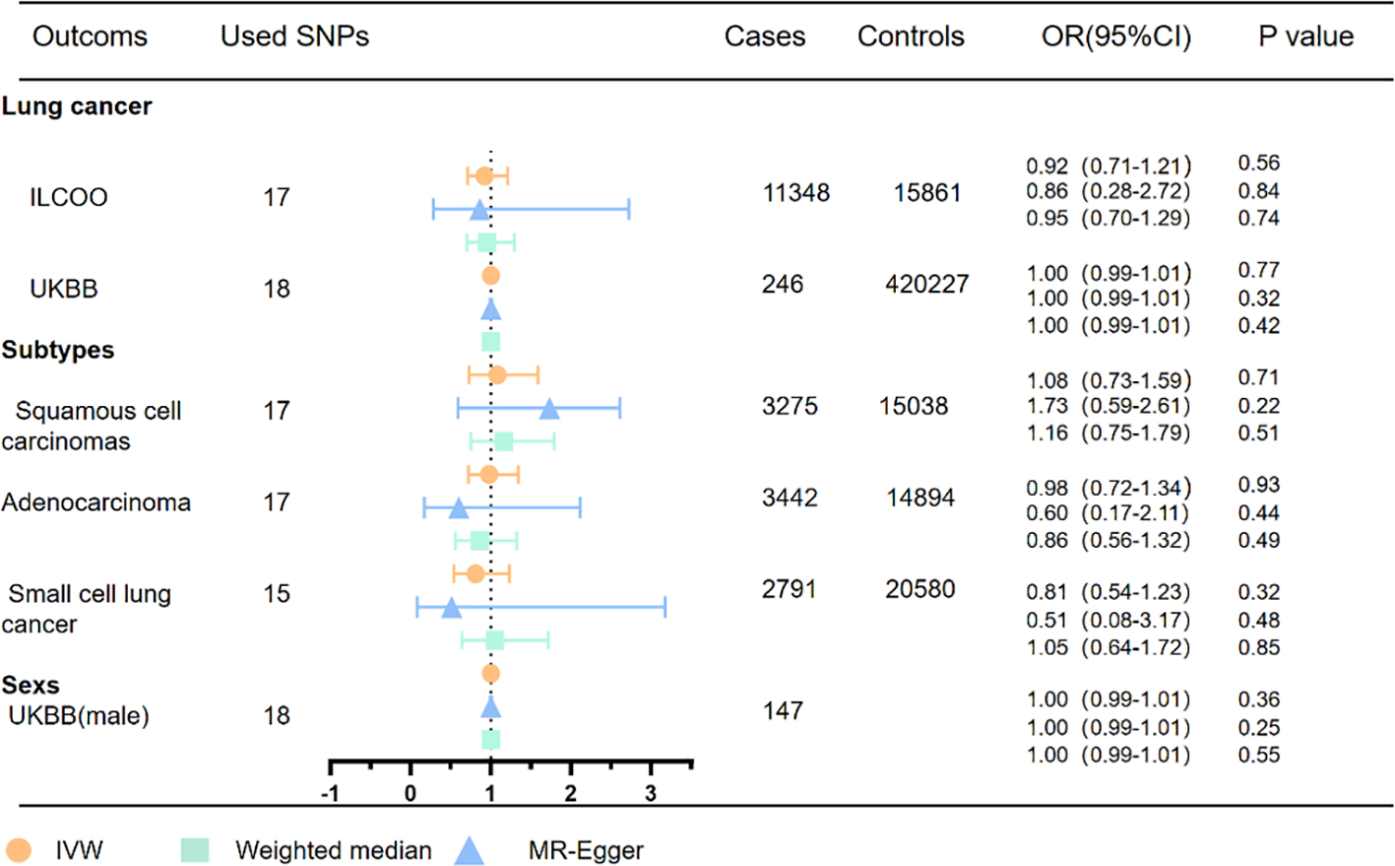
MR analyses of OSA with risk of lung cancer and pathological subtypes. OSA, obstructive sleep apnea; OR, odds ratio; CI, confidence interval; IVW, inverse variance weighted method; ILCCO, International Lung Cancer Consortium.

Further subgroup analysis based on the length of follow-up suggested that studies with a median follow-up of 7 years or longer had a higher pooled HR of 1.28 (95% CI 1.07–1.54) with reduced heterogeneity (I^2^ = 44%, p = 0.15), as shown in **Figure 4**. Moreover, a sensitivity analysis conducted exclusively on studies adjusting for smoking consistently yielded similar results. In particular, three studies that adjusted for smoking reported an HR of 1.32 (95% CI 1.26–1.37, P=0.07, I^2^=63%).

### 3.4 Certainty of evidence

Due to the limitations of observational studies, the quality of initial evidence for observational studies in Grading of Recommendations Assessment is low. The evidence quality regarding the lung cancer incidence was determined to be of low quality. This downgrade occurred due to two factors. First, there was a significant statistical heterogeneity, indicating variations in study findings. Second, there was a potential for publication bias, which could not be adequately assessed due to a limited number of studies. Additionally, it should be noted that the evidence quality for our specific subgroup of studies, characterized by a mean follow-up duration of at least 7 years, is also low (**Supplementary Table 2**).

### 3.5 Genetic instruments

Following the criteria for genetic instrument selection, a total of 17 independent SNPs were chosen as instruments for analyzing OSA in the ILCCO database, while 18 independent SNPs were selected for the UK Biobank database. More detailed information about the instruments used for each exposure can be found in **Supplementary Tables 4**, **6**. Importantly, all F statistics for the instruments used in the MR analyses were greater than 10, indicating the presence of robust instrument variables (**Supplementary Table 4**). **Supplementary Figure 1** illustrates the process of IV selection conducted in this study.

### 3.6 Causal effects of OSA on lung cancer and pathological subtypes

Within the scope of OSA genetic instrumental variables, the results of the univariate MR analysis indicate no significant association between OSA and an increased risk of total lung cancer, as demonstrated in **Figure 4** and confirmed by the ILCCO database (IVW: OR = 0.92, 95% CI 0.71–1.21, P =0.56). These findings are consistent with the results obtained from the UK Biobank database (IVW: OR =1.00, 95%CI 0.99–1.01, P =0.77). Moreover, the examination of specific pathological subtypes of lung cancer did not reveal any significant associations between genetically predicted OSA and the risk of lung adenocarcinoma (IVW: OR = 0.98, 95% CI 0.72–1.34, P =0.93), small cell lung cancer (IVW: OR =0.81, 95% CI 0.54–1.23, P = 0.32), and lung squamous cell carcinomas (IVW: OR = 1.08, 95% CI 0.73–1.59, P = 0.71).

Consistently, the effect estimator exhibited a consistent direction in the weighted median analysis. While the analysis of total lung cancer and lung squamous cell carcinomas in the ILCCO database identified an outlier through MRPRESSO, excluding this outlier variant did not alter the results. Heterogeneity tests indicated the presence of some heterogeneity among the individual SNP effect estimates. Furthermore, the MR-Egger intercept tests did not detect any horizontal pleiotropy in any of the conducted MR analyses (**Supplementary Figures 3-5**) (**Supplementary Tables 7**, **8**).

### 3.7 Causal effect from OSA on potential lung cancer risk factors

Utilizing the IVW method, we conducted a study to investigate the potential impact of various lung cancer-related factors on the association between genetically determined OSA and lung cancer. Our analysis focused on assessing the relationship between OSA and several risk factors for lung cancer, such as smoking, COPD, and body mass index, as shown in **Supplementary Figure 2**. However, our investigation did not reveal any supportive evidence indicating a causal relationship between OSA and these potential risk factors for lung cancer, as summarized in **Supplementary Table 9**.

## 4 Discussion

This paragraph presents the findings of a comprehensive meta-analysis involving a combined cohort of 6,589,725 patients. The analysis revealed that individuals with OSA had an incidence of lung cancer that was 11% higher compared to those without OSA. The study emphasized the significance of long-term monitoring in studies focused on detecting lung cancer incidence, as this difference was observed over a follow-up period of more than 7 years. Both OSA and lung cancer are chronic disease, and the time interval between their onset and detection can stretch out for several years. To establish a link between the physiological effects of OSA and the development of cancer, it is crucial for OSA to be present, even if undiagnosed, several years preceding the diagnosis of cancer. For instance, the research findings indicated that the typical time for squamous cell lung carcinoma to reach a diagnostic size is 8 years (35).

We then employed a two-sample MR approach to comprehensively investigate the potential causal effect of OSA on the incidence of lung cancer. Based on our analysis, the evidence produced inconclusive results regarding the existence of a causal relationship between genetically predicted OSA and the lung cancer. Among various lung cancer cell lines, H520 (human squamous cell lung cancer) demonstrated the most significant proliferation in response to hypoxemia. Different subtypes of lung cancer may respond differently to hypoxia (36). Nevertheless, our analysis did not reveal any notable correlations between genetically predicted OSA and the specific pathological subtypes of lung cancer, such as lung adenocarcinoma, small cell lung cancer, and lung squamous cell carcinomas. The findings of the meta-analysis depicting a significant increase in lung cancer risk seem to be at odds with the outcomes derived from the MR analysis. This inconsistency can be attributed to the inherent disparities and constraints inherent in observational studies. The majority of the studies included in this analysis are retrospective, which inherently brings limitations in the quality of the collected data. Maximize the sample size and enhance the accuracy of analyses related to specific cancer sites, numerous epidemiological studies have utilized national insurance health databases (5, 8, 31–33). These databases are used to identify individuals with OSA by examining recorded diagnostic codes. While these resources are valuable, they also introduce potential biases. Within these databases, certain confounding factors, such as obesity and smoking status, are of great importance but frequently unattainable. The insufficient management of these influential factors can significantly influence the interpretation of findings and produce varying repercussions across different studies, contingent upon the prevalence of these risk factors. The utilization of administrative databases to identify individuals with OSA introduces the potential for selection bias and the misclassification of exposure (37). Hence, the control group categorized as “unexposed” due to the lack of an OSA diagnosis might encompass numerous patients who are actually undiagnosed with OSA. This issue becomes more prominent in clinical settings where patients often possess risk factors for OSA, such as obesity. Conversely, individuals who have received a diagnosis of OSA may not adequately represent the entire population of OSA patients. Overcoming these limitations can be achieved through studies conducted in community settings, utilizing objective indicators to determine the presence of OSA. However, it is essential to acknowledge that such studies require significant resources and are consequently constrained in terms of sample size (38).

**Figure 5:**
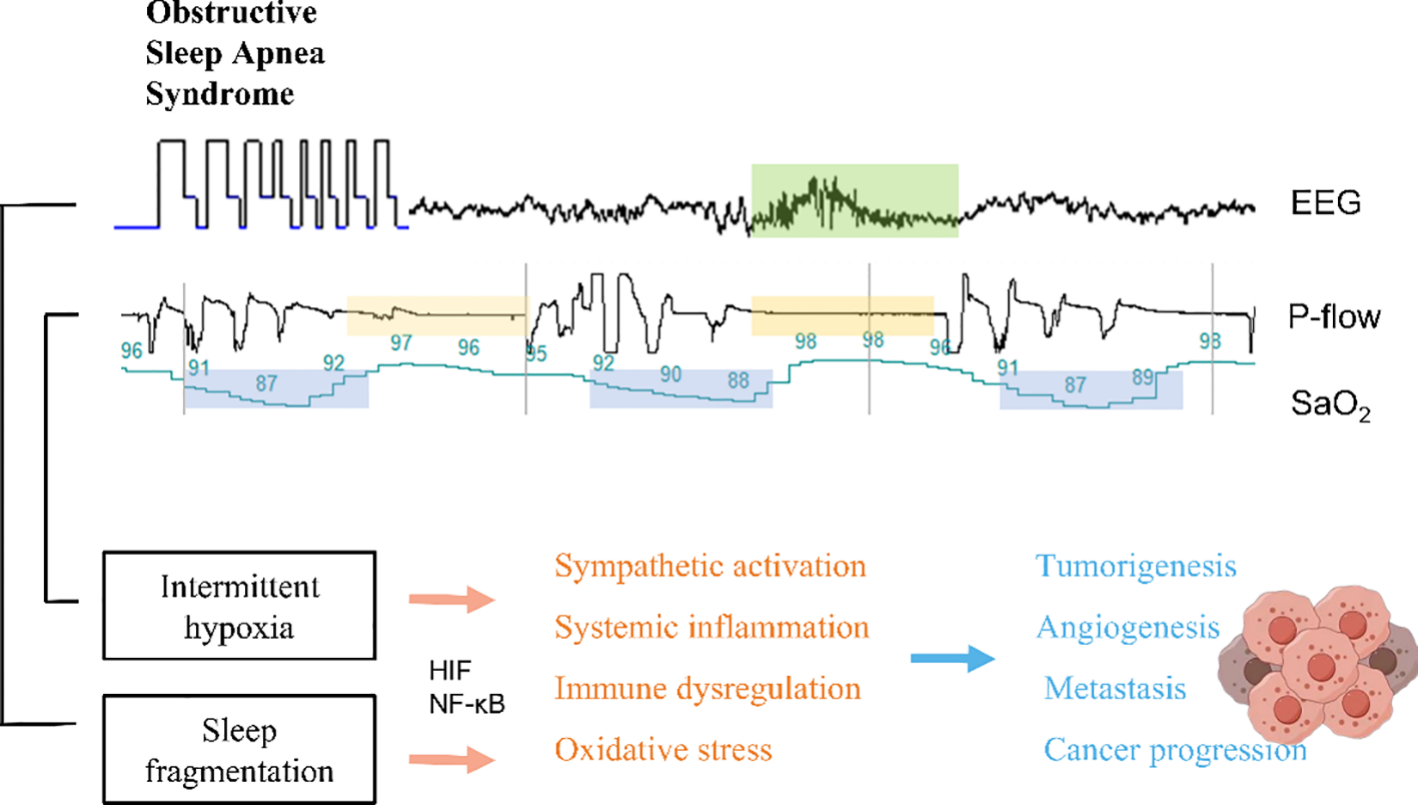
Potential mechanisms affected by either sleep disruption or intermittent hypoxia in the context of OSA.

Although our findings suggest no causality between OSA and lung cancer incidence, it is possible that OSA may have an impact on the progression of lung cancer. At both the biological and behavioral levels, there is widespread acceptance of the numerous underlying pathways connecting OSA and lung cancer. One potential pathway is the effect of OSA on sleep fragmentation. Sleep fragmentation, a covert form of sleep deprivation, may contribute to the development of cancer. Notably, research indicates that sleep fragmentation can stimulate the migration of macrophages to the artery, resulting in metabolic alterations that potentially facilitate the progression of malignancy. Furthermore, the influence of sleep fragmentation on tumorigenesis and advancement could be attributed to the disruption of the tightly linked biological clock associated with sleep disorders (39). Another potential pathway is the effect of intermittent hypoxia. Intermittent hypoxia has been associated with tumor growth and progression. Hypoxia-inducible factor (HIF)-1 and metabolic pathway-related molecules in lung cancer cells undergo significant changes under hypoxic conditions, playing a crucial role in the response of lung cancer cells to hypoxia. Animal experiments have revealed that in a mouse model of melanoma-induced lung metastasis, the OSA model not only promotes melanoma growth but also induces alterations in tumor-related macrophages, increasing invasiveness and facilitating the metastatic process. Intermittent hypoxia, resulting from cycles of hypoxia and reoxygenation, induces the generation of reactive oxygen species (ROS) or oxygen free radicals, triggering an activation of the oxidative stress response. This leads to an imbalance in the body’s oxidation and antioxidant substances, thereby causing acute and chronic deterioration of cellular function and structure, DNA damage, and genomic instability. Consequently, these processes promote cell proliferation and malignant transformation. Moreover, oxidative stress-induced nuclear factor-kappa B (NF-κB) activation can contribute to an increased cancer incidence. Patients with OSA experience both systemic and local inflammatory reactions. The disrupted balance of antioxidant production and increased ROS production further elevate levels of inflammatory factors such as tumor necrosis factor-alpha (TNF-α), interleukin-6 (IL-6), and IL-8, all of which can further stimulate NF-κB activation, thereby promoting cancer occurrence and progression (3, 34, 40). Intermittent hypoxia (IH) in OSA promotes cancer progression by upregulating HIF-1α and transforming growth factor β1 (TGF-β1), which alter cytokine levels, increase TNF-α and IL-10, and decrease IL-17, suppressing antitumor immunity (41). IH also elevates paraspeckle protein-1 (PSPC1), activating the TGF-β-SMAD pathway, and promoting epithelial-mesenchymal transition (EMT) and cancer stem cell (CSC)-like features (42–44). Furthermore, IH induces an immunosuppressive phenotype in monocytes, impairing NK cell function, and increases soluble immune checkpoints (PD-1/PD-L1) and midkine, facilitating immune evasion and lymphangiogenesis (45). These mechanisms collectively enhance tumor aggressiveness and progression in OSA patients. In addition, cancer can be influenced by various indirect pathways associated with behavior pattern of living, including smoking and obesity (**Figure 5**).

Our study possesses several notable strengths. Firstly, while traditional observational studies and RCTs serve as prominent research methods, the former is prone to bias, confounding factors, and reverse causality when investigating causal relationships. In contrast, MR draws upon the concept of instrumental variable methods utilized in economics, skillfully addressing issues of interference in causal inference. Notably, MR offers an effective alternative to the limitations associated with RCT research. Secondly, we conducted our causal estimation using two extensive databases, ensuring the consistency of our findings and providing reliable causal inference. This robustness strengthens the reliability of our results. Thirdly, while our study reveals an association between OSA and lung cancer, the lack of conclusive evidence for a causal relationship suggests that further research is needed to fully understand the public health implications. Current findings are not sufficient to directly influence public health policies, but they provide a foundation for future investigations. Our findings suggest that there may be limited value in enhancing lung cancer screening solely in patients with genetically predicted OSA. Therefore, it is crucial to direct greater attention towards uncovering the correlation between environment-induced OSA and lung carcinogenesis, as well as exploring the connection between OSA and the prognosis of lung cancer.

However, our study has certain limitations that need to be acknowledged. Firstly, It is important to highlight that not all of the studies analyzed in our research accounted for smoking status. This is due to a lack of available data on smoking habits among participants in certain studies. However, it is noteworthy that Kendzerska et al. conducted a sensitivity analysis, which included smoking status as a subgroup. Secondly, because only summary-level statistics were available, we encountered limitations in conducting stratified analyses involving age, as well as other covariates like specific subtypes of lung cancer, gender, and smoking status. One possible limitation to our MR analysis was the possibility of potential overlap, due to restriction to European populations (40). Although we utilized data from both the UK Biobank and ILCCO databases. It is important to note that OSA is a binary exposure, and the instrumental variable estimate we obtained represents the average causal estimate in individuals influenced by the genetic variants used to determine OSA presence or absence (46). When applying MR analysis to binary exposures, it is possible to obtain relative risk values that are not precisely identifiable but have identifiable boundaries. Additionally, it is worth mentioning that all the GWAS data used in our study were derived from European populations. Therefore, it is crucial to examine whether our findings remain consistent in other populations.

## 5 Conclusion

In our research, we discovered a clear connection between OSA and an increased likelihood of developing lung cancer, as observed in population-based cohort studies. However, it is important to note that the study using MR did not establish a direct cause-and-effect relationship between OSA and lung cancer. The significant association seen in the observational studies may be influenced by biases inherent in these types of studies, such as inaccurate diagnoses of OSA, inadequate adjustment for factors that may confound the results, and other potential limitations. Additionally, to validate our findings and provide more definitive evidence regarding the association between OSA and lung cancer, it would be advantageous to conduct well-orchestrated epidemiological studies and MR studies that incorporate a larger number of instrumental variables and samples. This would help strengthen the reliability of our findings and provide more compelling insights into the relationship between OSA and lung cancer.

## Data availability statement

Publicly available datasets were analyzed in this study. This data can be found here: ILCCO, FinnGen (https://www.finngen.fi/en), GSCAN GIANT, and the UK Biobank study.

## Ethics statement

The cited genome-wide association studies incorporated in this paper were granted approval by the appropriate review board, and all participants had provided written informed consent.

## Author contributions

JY: Writing – original draft. RD: Conceptualization, Writing – original draft. QL: Conceptualization, Data curation, Formal analysis, Writing – original draft. RM: Validation, Software, Writing – original draft, Writing – review & editing. PZ: Project administration, Writing – review & editing. TF: Supervision, Validation, Writing – review & editing.
