# Supplementary material for: Association between obstructive sleep apnea and risk of lung cancer: findings from a collection of cohort studies and Mendelian randomization analysis
Source: Front Oncol. 2024 Jul 12;14:1346809. doi: 10.3389/fonc.2024.1346809 (PMC11272613; doi:10.3389/fonc.2024.1346809)
Supplement: Supplementary file 1 [file DataSheet_1.docx]

**DATA SUPPLEMENT**

**Association between obstructive sleep apnea and risk of lung cancer: results from a pool of cohort studies and Mendelian randomization analysis**

**List of Supplemental Materials**

**Table S1.** PRISMA Checklist

**Table S2.** Evaluation of quality of pooled evidence using the Grading of Recommendations Assessment, Development and Evaluation (GRADE) framework

**Table S3.** STROBE-MR checklist of recommended items to address in reports of Mendelian randomization studies

**Table S4**. Information on instrumental variables of OSA

**Table S5.** Information on GWAS summary statistics

**Table S6.** Summary information on lung cancer-related phenotypes for the 20 genome-wide significant SNPs associated with OSA

**Table S7.** Associations of genetically predicted OSA with risk of lung cancer in sensitivity analyses

**Table S8**. Associations of genetically predicted OSA with risk of lung cancer in the MR-PRESSO analysis

**Table S9**. MR estimates of the associations from OSA on common risk factors

**Figure S1.** Flowchart of the SNP selection process for all univariate MR analyses.

**Figure S2.** The MR association between OSA and lung cancer was not violated through pleiotropic pathways relating to lung cancer.

**Figure S3.** Scatter plot of SNPs associated with OSA and their risk of lung cancer. (A) total lung cancer (B) lung adenocarcinoma (C) lung squamous cell carcinoma (D) small cell lung carcinoma (E) total lung cancer(UKB-both sex) (F) total lung cancer(UKB-male)

**Figure S4.** Funnel plot of SNPs associated with OSA and their risk of lung cancer. (A) total lung cancer (B) lung adenocarcinoma (C) lung squamous cell carcinoma (D) small cell lung carcinoma (E) total lung cancer(UKB-both sex) (F) total lung cancer(UKB-male)

**Figure S5.** Leave-one-out of SNPs associated with OSA and their risk of lung cancer. (A) total lung cancer (B) lung adenocarcinoma (C) lung squamous cell carcinoma (D) small cell lung carcinoma (E) total lung cancer(UKB-both sex) (F) total lung cancer(UKB-male)

**Supplemental Table S1: PRISMA Checklist**

| **Section and**  **Topic** | **Item #** | **Checklist item** |
| --- | --- | --- |
| **TITLE** | | |
| Title | 1 | Identify the report as a systematic review. |
| **ABSTRACT** | | |
| Abstract | 2 | See the PRISMA 2020 for Abstracts checklist. |
| **INTRODUCTION** | | |
| Rationale | 3 | Describe the rationale for the review in the context of existing knowledge. |
| Objectives | 4 | Provide an explicit statement of the objective(s) or question(s) the review addresses. |
| **METHODS** | | |
| Eligibility criteria | 5 | Specify the inclusion and exclusion criteria for the review and how studies were grouped for the syntheses. |
| Information  sources | 6 | Specify all databases, registers, websites, organisations, reference lists and other sources searched or consulted to identify studies. Specify the date when each source was last searched or consulted. |
| Search strategy | 7 | Present the full search strategies for all databases, registers and websites, including any filters and limits used. |
| Selection process | 8 | Specify the methods used to decide whether a study met the inclusion criteria of the review, including how many reviewers screened each record and each report retrieved, whether they worked independently, and if applicable, details of automation tools used in the process. |
| Data collection  process | 9 | Specify the methods used to collect data from reports, including how many reviewers collected data from each report, whether they worked  independently, any processes for obtaining or confirming data from study investigators, and if applicable, details of automation tools used in the process. |
| Data items | 10a | List and define all outcomes for which data were sought. Specify whether all results that were compatible with each outcome domain in each study were sought (e.g. for all measures, time points, analyses), and if not, the methods used to decide which results to collect. |
|  | 10b | List and define all other variables for which data were sought (e.g. participant and intervention characteristics, funding sources). Describe any assumptions made about any missing or unclear information. |
| Study risk of bias assessment | 11 | Specify the methods used to assess risk of bias in the included studies, including details of the tool(s) used,how many reviewers assessed each study and whether they worked independently, and if applicable, details of automation tools used in the process. |
| Effect measures | 12 | Specify for each outcome the effect measure(s) (e.g. risk ratio, mean difference) used in the synthesis or presentation of results. |

| **Section and**  **Topic** | **Item #** | **Checklist item** |
| --- | --- | --- |
| Synthesis  methods | 13a | Describe the processes used to decide which studies were eligible for each synthesis (e.g. tabulating the study intervention characteristics and comparing against the planned groups for each synthesis (item #5)). |
|  | 13b | Describe any methods required to prepare the data for presentation or synthesis, such as handling of missing summary statistics, or data conversions. |
|  | 13c | Describe any methods used to tabulate or visually display results of individual studies and syntheses. |
|  | 13d | Describe any methods used to synthesize results and provide a rationale for the choice(s). If meta-analysis was performed, describe the model(s), method(s) to identify the presence and extent of statistical heterogeneity, and software package(s) used. |
|  | 13e | Describe any methods used to explore possible causes of heterogeneity among study results (e.g. subgroup analysis, meta-regression). |
|  | 13f | Describe any sensitivity analyses conducted to assess robustness of the synthesized results. |
| Reporting bias  assessment | 14 | Describe any methods used to assess risk of bias due to missing results in a synthesis (arising from reporting biases). |
| Certainty  assessment | 15 | Describe any methods used to assess certainty (or confidence) in the body of evidence for an outcome. |
| **RESULTS** | | |
| Study selection | 16a | Describe the results of the search and selection process, from the number of records identified in the search to the number of studies included in the review, ideally using a flow diagram. |
|  | 16b | Cite studies that might appear to meet the inclusion criteria, but which were excluded, and explain why they were excluded. |
| Study  characteristics | 17 | Cite each included study and present its characteristics. |
| Risk of bias in  studies | 18 | Present assessments of risk of bias for each included study. |
| Results of  individual studies | 19 | For all outcomes, present, for each study: (a) summary statistics for each group (where appropriate) and (b) an effect estimate and its precision (e.g. confidence/credible interval), ideally using structured tables or plots. |
| Results of  syntheses | 20a | For each synthesis, briefly summarise the characteristics and risk of bias among contributing studies. |
|  | 20b | Present results of all statistical syntheses conducted. If meta-analysis was done, present for each the summary estimate and its precision (e.g. confidence/credible interval) and measures of statistical heterogeneity. If comparing groups, describe the direction of the effect. |
|  | 20c | Present results of all investigations of possible causes of heterogeneity among study results. |
|  | 20d | Present results of all sensitivity analyses conducted to assess the robustness of the synthesized results. |
| Reporting biases | 21 | Present assessments of risk of bias due to missing results (arising from reporting biases) for each synthesis assessed. |
| Certainty of | 22 | Present assessments of certainty (or confidence) in the body of evidence for each outcome assessed. |

| **Section and**  **Topic** | **Item #** | **Checklist item** |
| --- | --- | --- |
| evidence |  |  |
| **DISCUSSION** | | |
| Discussion | 23a | Provide a general interpretation of the results in the context of other evidence. |
|  | 23b | Discuss any limitations of the evidence included in the review. |
|  | 23c | Discuss any limitations of the review processes used. |
|  | 23d | Discuss implications of the results for practice, policy, and future research. |
| **OTHER INFORMATION** | | |
| Registration and protocol | 24a | Provide registration information for the review, including register name and registration number, or state that the review was not registered. |
|  | 24b | Indicate where the review protocol can be accessed, or state that a protocol was not prepared. |
|  | 24c | Describe and explain any amendments to information provided at registration or in the protocol. |
| Support | 25 | Describe sources of financial or non-financial support for the review, and the role of the funders or sponsors in the review. |
| Competing  interests | 26 | Declare any competing interests of review authors. |
| Availability of  data, code and  other materials | 27 | Report which of the following are publicly available and where they can be found: template data collection forms; data extracted from included studies; data used for all analyses; analytic code; any other materials used in the review. |

E, Bossuyt PM, Boutron I, Hoffmann TC, Mulrow CD, et al. The PRISMA 2020 statement: an updated guideline for reporting systematic reviews. BMJ 2021;372:n71. doi: 10.1136/bmj.n71

For more information, visit: http://www.prisma-statement.org/

**Table S2: Evaluation of quality of pooled evidence using the Grading of Recommendations Assessment, Development and Evaluation (GRADE) framework**

| Outcomes | Pooled outcomes (95% CI) | No. of patients (no. of included studies) | Statistical  heterogeneity | Quality of evidence (GRADE) |
| --- | --- | --- | --- | --- |
| Lung cancer incidence | 1.11 (0.93-1.33) | 6,589,725  (6 studies) | I² = 95% | ⊕⦵⦵⦵a,b |
| Lung cancer incidence in  subgroup with at least seven years of mean follow-up duration | 1.32 (1.27-1.37) | 1,496,615  (3 studies) | I² = 44% | ⊕⦵⦵⦵a |
| aDowngraded by one level for the possibility of publication bias which could not be evaluated due to insufficient studies. bDowngraded by one level for substantial statistical heterogeneity.  No outcomes were downgraded for risk of bias (since all studies ≥5 based on NOS), imprecision (since large sample size and narrow confidence interval), or indirectness of evidence. | | | | |

**Table S3:STROBE-MR checklist of recommended items to address in reports of Mendelian randomization studies**^1^ ^2^

| **Item No.** | **Section** | **Checklist item** | **Relevant text from manuscript** |
| --- | --- | --- | --- |
| 1 | **TITLE and ABSTRACT** | Indicate Mendelian randomization (MR) as the study’s design in the title and/or the abstract if that is a main purpose of the study |  |
|  | **INTRODUCTION** |  |  |
| 2 | **Background** | Explain the scientific background and rationale for the reported study. What is the exposure? Is a potential causal relationship between exposure and outcome plausible? Justify why MR is a helpful method to address the study question |  |
| 3 | **Objectives** | State specific objectives clearly, including pre-specified causal hypotheses (if any). State that MR is a method that, under specific assumptions, intends to estimate causal effects |  |
|  | **METHODS** |  |  |
| 4 | **Study design and data sources** | Present key elements of the study design early in the article. Consider including a table listing sources of data for all phases of the study. For each data source contributing to the analysis, describe the following: |  |
|  | a) | Setting: Describe the study design and the underlying population, if possible. Describe the setting, locations, and relevant dates, including periods of recruitment, exposure, follow-up, and data collection, when available. |  |
|  | b) | Participants: Give the eligibility criteria, and the sources and methods of selection of participants. Report the sample size, and whether any power or sample size calculations were carried out prior to the main analysis |  |
|  | c) | Describe measurement, quality control and selection of genetic variants |  |
|  | d) | For each exposure, outcome, and other relevant variables, describe methods of assessment and diagnostic criteria for diseases |  |
|  | e) | Provide details of ethics committee approval and participant informed consent, if relevant |  |
| 5 | **Assumptions** | Explicitly state the three core IV assumptions for the main analysis (relevance, independence and exclusion restriction) as well assumptions for any additional or sensitivity analysis |  |
| 6 | **Statistical methods: main analysis** | Describe statistical methods and statistics used |  |
|  | a) | Describe how quantitative variables were handled in the analyses (i.e., scale, units, model) |  |
|  | b) | Describe how genetic variants were handled in the analyses and, if applicable, how their weights were selected |  |
|  | c) | Describe the MR estimator (e.g. two-stage least squares, Wald ratio) and related statistics. Detail the included covariates and, in case of two-sample MR, whether the same covariate set was used for adjustment in the two samples |  |
|  | d) | Explain how missing data were addressed |  |
|  | e) | If applicable, indicate how multiple testing was addressed |  |
| 7 | **Assessment of assumptions** | Describe any methods or prior knowledge used to assess the assumptions or justify their validity |  |
| 8 | **Sensitivity analyses and additional analyses** | Describe any sensitivity analyses or additional analyses performed (e.g. comparison of effect estimates from different approaches, independent replication, bias analytic techniques, validation of instruments, simulations) |  |
| 9 | **Software and pre-registration** |  |  |
|  | a) | Name statistical software and package(s), including version and settings used |  |
|  | b) | State whether the study protocol and details were pre-registered (as well as when and where) |  |
|  | **RESULTS** |  |  |
| 10 | **Descriptive data** |  |  |
|  | a) | Report the numbers of individuals at each stage of included studies and reasons for exclusion. Consider use of a flow diagram |  |
|  | b) | Report summary statistics for phenotypic exposure(s), outcome(s), and other relevant variables (e.g. means, SDs, proportions) |  |
|  | c) | If the data sources include meta-analyses of previous studies, provide the assessments of heterogeneity across these studies |  |
|  | d) | For two-sample MR:  i.  Provide justification of the similarity of the genetic variant-exposure associations between the exposure and outcome samples  ii.  Provide information on the number of individuals who overlap between the exposure and outcome studies |  |
| 11 | **Main results** |  |  |
|  | a) | Report the associations between genetic variant and exposure, and between genetic variant and outcome, preferably on an interpretable scale |  |
|  | b) | Report MR estimates of the relationship between exposure and outcome, and the measures of uncertainty from the MR analysis, on an interpretable scale, such as odds ratio or relative risk per SD difference |  |
|  | c) | If relevant, consider translating estimates of relative risk into absolute risk for a meaningful time period |  |
|  | d) | Consider plots to visualize results (e.g. forest plot, scatterplot of associations between genetic variants and outcome versus between genetic variants and exposure) |  |
| 12 | **Assessment of assumptions** |  |  |
|  | a) | Report the assessment of the validity of the assumptions |  |
|  | b) | Report any additional statistics (e.g., assessments of heterogeneity across genetic variants, such as *I^2^*, Q statistic or E-value) |  |
| 13 | **Sensitivity analyses and additional analyses** |  |  |
|  | a) | Report any sensitivity analyses to assess the robustness of the main results to violations of the assumptions |  |
|  | b) | Report results from other sensitivity analyses or additional analyses |  |
|  | c) | Report any assessment of direction of causal relationship (e.g., bidirectional MR) |  |
|  | d) | When relevant, report and compare with estimates from non-MR analyses |  |
|  | e) | Consider additional plots to visualize results (e.g., leave-one-out analyses) |  |
|  | **DISCUSSION** |  |  |
| 14 | **Key results** | Summarize key results with reference to study objectives |  |
| 15 | **Limitations** | Discuss limitations of the study, taking into account the validity of the IV assumptions, other sources of potential bias, and imprecision. Discuss both direction and magnitude of any potential bias and any efforts to address them |  |
| 16 | **Interpretation** |  |  |
|  | a) | Meaning: Give a cautious overall interpretation of results in the context of their limitations and in comparison with other studies |  |
|  | b) | Mechanism: Discuss underlying biological mechanisms that could drive a potential causal relationship between the investigated exposure and the outcome, and whether the gene-environment equivalence assumption is reasonable. Use causal language carefully, clarifying that IV estimates may provide causal effects only under certain assumptions |  |
|  | c) | Clinical relevance: Discuss whether the results have clinical or public policy relevance, and to what extent they inform effect sizes of possible interventions |  |
| 17 | **Generalizability** | Discuss the generalizability of the study results (a) to other populations, (b) across other exposure periods/timings, and (c) across other levels of exposure |  |
|  | **OTHER INFORMATION** |  |  |
| 18 | **Funding** | Describe sources of funding and the role of funders in the present study and, if applicable, sources of funding for the databases and original study or studies on which the present study is based |  |
| 19 | **Data and data sharing** | Provide the data used to perform all analyses or report where and how the data can be accessed, and reference these sources in the article. Provide the statistical code needed to reproduce the results in the article, or report whether the code is publicly accessible and if so, where |  |
| 20 | **Conflicts of Interest** | All authors should declare all potential conflicts of interest |  |

This checklist is copyrighted by the Equator Network under the Creative Commons Attribution 3.0 Unported (CC BY 3.0) license.

1. Skrivankova VW, Richmond RC, Woolf BAR, Yarmolinsky J, Davies NM, Swanson SA, et al. Strengthening the Reporting of Observational Studies in Epidemiology using Mendelian Randomization (STROBE-MR) Statement. JAMA. 2021;under review.

2. Skrivankova VW, Richmond RC, Woolf BAR, Davies NM, Swanson SA, VanderWeele TJ, et al. Strengthening the Reporting of Observational Studies in Epidemiology using Mendelian Randomisation (STROBE-MR): Explanation and Elaboration. BMJ. 2021;375:n2233.

**Table S4**. Information on instrumental variables of OSA

| **RSID** | **CHR** | **Position** | **A1** | **A2** | **EAF** | **Beta** | **SE** | **P** | **R^2^** | **F-statistic** |
| --- | --- | --- | --- | --- | --- | --- | --- | --- | --- | --- |
| rs1228509 | 1 | 96564588 | C | A | 0.6562 | 0.0470 | 0.0081 | 6.41E-09 | 1.00E-03 | 376 |
| rs76229479 | 2 | 102507453 | C | A | 0.0987 | -0.0779 | 0.0131 | 2.64E-09 | 1.08E-03 | 407 |
| rs114106239 | 3 | 132779050 | T | C | 0.0371 | -0.1205 | 0.0211 | 1.19E-08 | 1.04E-03 | 391 |
| rs13114985 | 4 | 47302092 | G | T | 0.3382 | 0.0456 | 0.0081 | 2.01E-08 | 9.34E-04 | 351 |
| rs60700772 | 6 | 7746430 | C | T | 0.2212 | 0.0516 | 0.0092 | 2.20E-08 | 9.21E-04 | 346 |
| rs11981973 | 7 | 69980355 | G | A | 0.1815 | 0.0612 | 0.0098 | 4.72E-10 | 1.12E-03 | 420 |
| rs679880 | 9 | 76652155 | A | G | 0.7450 | 0.0495 | 0.0088 | 2.53E-08 | 9.33E-04 | 351 |
| rs10986730 | 9 | 125375595 | T | C | 0.5241 | -0.0467 | 0.0076 | 1.16E-09 | 1.09E-03 | 411 |
| rs113955098 | 10 | 18302297 | A | G | 0.0674 | -0.0994 | 0.0158 | 3.38E-10 | 1.24E-03 | 468 |
| rs61873510 | 10 | 100866753 | T | G | 0.3010 | 0.0464 | 0.0084 | 3.28E-08 | 9.07E-04 | 341 |
| rs6484367 | 11 | 28365912 | A | G | 0.5026 | 0.0468 | 0.0076 | 1.13E-09 | 1.10E-03 | 413 |
| rs59333125 | 12 | 56632124 | C | A | 0.0807 | -0.0815 | 0.0143 | 1.32E-08 | 9.88E-04 | 372 |
| rs10507084 | 12 | 97359374 | T | C | 0.1794 | 0.0647 | 0.0099 | 8.23E-11 | 1.23E-03 | 464 |
| rs2016950 | 12 | 107586546 | T | C | 0.1579 | -0.0586 | 0.0106 | 4.13E-08 | 9.15E-04 | 344 |
| rs2370982 | 14 | 79424334 | T | C | 0.2381 | 0.0515 | 0.0089 | 7.97E-09 | 9.63E-04 | 362 |
| rs11075985 | 16 | 53771295 | A | C | 0.4287 | 0.0820 | 0.0077 | 2.15E-26 | 3.30E-03 | 1243 |
| rs13333522 | 16 | 73774720 | G | C | 0.5266 | 0.0423 | 0.0077 | 4.06E-08 | 8.94E-04 | 336 |
| rs10423928 | 19 | 45679046 | A | T | 0.2585 | -0.0509 | 0.0088 | 7.55E-09 | 9.97E-04 | 375 |
| rs4809902 | 20 | 52363114 | C | G | 0.2284 | -0.0556 | 0.0092 | 1.51E-09 | 1.09E-03 | 411 |
| rs140896965 | 20 | 59073311 | T | C | 0.0490 | -0.1125 | 0.0184 | 1.11E-09 | 1.18E-03 | 444 |

CHR, chromosome; A1, effect allele; A2,baseline allele; EAF, effect allele frequency; SE, standard error; SNP, single nucleotide polymorphism; OSA, obstructive sleep apnea; Beta value is equal to log (OR).

**Table S5**. Information on GWAS summary statistics

| **Data source** | **Phenotypes** | **Population** | **Sample size**  **(Cases /Controls)** | | | **Covariates adjusted in GWAS** |
| --- | --- | --- | --- | --- | --- | --- |
| Finngen | Obstructive sleep apnea | European | 38998 | | 336659 | Age, sex, and ten genetic principal components |
| ILCOO | Total lung cancer  Adenocarcinoma  Squamous cell carcinomas  Small cell lung cancer | European | 11348  3442  3275  2791 | 15861  14894  15038  20580 | | Not reported |
| UKBB | Total lung cancer(both sex)  Total lung cancer(male)  COPD | European | 246  147  1605 | 420227  461328 | | Age, sex, and ten genetic principal components |
| GAINT | BMI | European | 681275 | | | Age, age-squared, sex, and principal  components 1-5 |
| GSCAN | Smoking initiation  Cigarettes smoked per day | European  European | 311629  249752 | | 321173 | Age, sex, and ten genetic principal components |
| GWAS and Sequencing Consortium of Alcohol and Nicotine use | Age Of Smoking Initiation | European | 341427 | | | Not reported |

BMI,body mass index; GIANT, Genetic Investigation of ANthropometric Traits;GWAS, genome-wide association study; GSCAN, GWAS & Sequencing Consortium of Alcohol and Nicotine; NA, not available; UKBB, UK Biobank;COPD, chronic obstructive airways disease.

**Table S6.** **Summary information on lung cancer-related phenotypes for the 20 genome-wide significant SNPs associated with OSA**

| **SNP** | **A1** | **A2** | **LICOO** | | | | **TRICL** | | | | **UKB** | | | |
| --- | --- | --- | --- | --- | --- | --- | --- | --- | --- | --- | --- | --- | --- | --- |
|  |  |  | **EAF** | **Beta** | **SE** | **P** | **EAF** | **Beta** | **SE** | **P** | **EAF** | **Beta** | **SE** | **P** |
| rs1228509 | C | A |  | 0.0251 | 0.0181 | 0.1849 |  | 0.0306 | 0.0304 | 0.3131 | 0.3464 | 8.57E-05 | 0.0001 | 0.3956 |
| rs76229479 | C | A |  | 0.0265 | 0.0285 | 0.3523 |  | 0.0637 | 0.0456 | 0.1627 | 0.1338 | -0.0002 | 0.0001 | 0.0923 |
| rs114106239 | T | C |  | 0.1059 | 0.0773 | 0.1567 |  |  |  |  | 0.0171 | 0.0001 | 0.0003 | 0.6202 |
| rs13114985 | G | T |  | 0.0102 | 0.0186 | 0.5865 |  | 0.0133 | 0.0299 | 0.6557 | 0.3777 | 9.48E-05 | 9.93E-05 | 0.3397 |
| rs60700772 | C | T |  | 0.0601 | 0.0229 | 0.0063 |  | 0.0453 | 0.0348 | 0.1935 | 0.2193 | -0.0001 | 0.0001 | 0.3684 |
| rs11981973 | G | A |  | -0.0296 | 0.0233 | 0.2276 |  | -0.0688 | 0.0391 | 0.0789 | 0.1680 | 0.0001 | 0.0001 | 0.4247 |
| rs679880 | A | G |  | 0.0057 | 0.0217 | 0.7979 |  | 0.0167 | 0.0364 | 0.6470 | 0.1925 | 0.0001 | 0.0001 | 0.2463 |
| rs10986730 | T | C |  | 0.0533 | 0.0188 | 0.0034 |  | 0.0583 | 0.0290 | 0.0447 | 0.4299 | 8.74E-05 | 9.69E-05 | 0.3673 |
| rs113955098 | A | G |  | -0.0525 | 0.0709 | 0.5153 |  |  |  |  |  |  |  |  |
| rs61873510 | T | G |  | -0.0076 | 0.0210 | 0.7256 |  | -0.0011 | 0.0330 | 0.9712 | 0.3277 | -0.0002 | 0.0001 | 0.0179 |
| rs6484367 | A | G |  | -0.0158 | 0.0178 | 0.3909 |  | -0.0006 | 0.0292 | 0.9825 | 0.4880 | 2.23E-05 | 9.63E-05 | 0.8164 |
| rs59333125 | C | A |  | 0.0088 | 0.0394 | 0.8271 |  | 0.1225 | 0.0587 | 0.0370 | 0.0728 | -4.07E-05 | 0.0001 | 0.8253 |
| rs10507084 | T | C |  | 0.0664 | 0.0351 | 0.0507 |  | 0.0617 | 0.0548 | 0.2600 | 0.0583 | -0.0002 | 0.0002 | 0.1951 |
| rs2016950 | T | C |  | -0.0013 | 0.0267 | 0.9607 |  | 0.0119 | 0.0431 | 0.7813 | 0.1307 | -0.0002 | 0.0001 | 0.0954 |
| rs2370982 | T | C |  | -0.0422 | 0.0208 | 0.0568 |  | -0.0754 | 0.0355 | 0.0338 | 0.2159 | -3.24E-05 | 0.0001 | 0.7850 |
| rs11075985 | A | C |  | 0.0011 | 0.0182 | 0.9489 |  | 0.0224 | 0.0294 | 0.4458 | 0.4228 | 7.85E-05 | 9.70E-05 | 0.4186 |
| rs13333522 | G | C |  |  |  |  |  |  |  |  |  |  |  |  |
| rs10423928 | A | T |  |  |  |  |  |  |  |  | 0.1929 | -9.40E-05 | 0.0001 | 0.4378 |
| rs4809902 | C | G |  |  |  |  |  |  |  |  | 0.2848 | 0.0001 | 0.0001 | 0.2637 |
| rs140896965 | T | C |  | 0.0418 | 0.0789 | 0.6092 |  | 0.1697 | 0.1312 | 0.1958 | 0.0065 | 0.0001 | 0.0006 | 0.7752 |

SNP, single nucleotide polymorphism; A1, effect allele; A2,baseline allele; EAF, effect allele frequency. All statistical tests were two-sided. A P-value < 5 ×10-8 was considered genome-wide significant and should be excluded to meet the the assumption that requires instruments to be associated with the outcome only through exposure

**Table S7.** Associations of genetically predicted OSA with risk of lung cancer in sensitivity analyses

| **Outcome** | **Source** | **SNPs**  **Used** | **Heterogeneity** | | | **Pleiotropy** | |
| --- | --- | --- | --- | --- | --- | --- | --- |
|  |  |  | **I ^2^** | **Cochrane’s Q** | **P** | **Intercept** | **Pintercept** |
| Total lung cancer | ILCOO | 17 | 47% | 30.38 | 0.01 | 0.002 | 0.941 |
| Adenocarcinoma | ILCOO | 17 | 6% | 17.09 | 0.379 | 0.029 | 0.435 |
| Squamous cell carcinomas | ILCOO | 17 | 5% | 27.39 | 0.037 | -0.056 | 0.235 |
| Small cell lung cancer | ILCOO | 15 | 39.23% | 23.04 | 0.05 | 0.027 | 0.612 |
| Total lung cancer(both sex) | UKBB | 18 | 5% | 17.98 | 0.395 | -7.346e-05 | 0.343 |
| Total lung cancer(male) | UKBB | 18 | 8% | 20.692 | 0.240 | -0.0001 | 0.426 |

SNP, single nucleotide polymorphism; ILCCO, International Lung Cancer Consortium ;UKBB, UK Biobank.

The *I2* statistic was used to present the heterogeneity among estimates for each SNPs in one analysis. The p value for the intercept in the MR-Egger regression was used present the pleiotropy (p<0.05).

**Table S8.** Associations of genetically predicted OSA with risk of lung cancer in the MR-PRESSO analysis

| **Outcome** | **Source** | **SNPs**  **Used** | **Outliers** | **Global**  **Test P-**  **value** | **Distortion**  **Test P-value** | **Estimate** | **P vaule** |
| --- | --- | --- | --- | --- | --- | --- | --- |
| Total lung cancer | ILCOO | 17 | 1 | 0.018 | 0.689 | -0.146 | 0.257 |
| Adenocarcinoma | ILCOO | 17 | 0 | 0.396 | NA | NA | NA |
| Squamous cell carcinomas | ILCOO | 17 | 1 | 0.04 | 0.570 | 0.214 | 0.199 |
| Small cell lung cancer | ILCOO | 15 | 0 | 0.381 | NA | NA | NA |
| Total lung cancer(both sex) | UKBB | 18 | 0 | 0.383 | NA | NA | NA |
| Total lung cancer(male) | UKBB | 18 | 0 | 0.248 | NA | NA | NA |

NA, not available; SNPs, singe nucleotide polymorphisms; ILCCO, International Lung Cancer Consortium ;UKBB, UK Biobank.

**Table S9.** MR estimates of the associations from OSA on common risk factors

| **Method** | **Number of SNPs** | **F-statistic** | **beta (95% CI)** | **P-value** |
| --- | --- | --- | --- | --- |
| OSA on Smoking initiation | | | | |
| IVW | 17 | 441 | 0.95(0.87,1.03) | 0.245 |
| Weighted median |  |  | 1.02(0.96,1.09) | 0.526 |
| MR-Egger |  |  | 1.16(0.85,1.59) | 0.354 |
| MR-Egger ***intercept*** |  |  | - | 0.213 |
| Q statistic |  |  | - | 5.94e-09 |
| OSA on Cigarettes smoked per day | | | | |
| IVW | 17 | 441 | 0.95(0.88,1.03) | 0.198 |
| Weighted median |  |  | 0.95(0.87,1.03) | 0.227 |
| MR-Egger |  |  | 0.99(0.73,1.35) | 0.981 |
| MR-Egger ***intercept*** |  |  | - | 0.767 |
| Q statistic |  |  | - | 0.052 |
| OSA on smoking Initiation | | | | |
| IVW | 17 | 441 | 1.01(0.97,1.04) | 0.610 |
| Weighted median |  |  | 1.00(0.96,1.05) | 0.854 |
| MR-Egger |  |  | 0.99(0.85,1.16) | 0.929 |
| MR-Egger ***intercept*** |  |  | - | 0.827 |
| Q statistic |  |  | - | 0.040 |
| OSA on BMI | | | | |
| IVW | 14 | 441 | 1.43(1.11,1.86) | 0.005 |
| Weighted median |  |  | 1.08(1.03,1.14) | 0.001 |
| MR-Egger |  |  | 4.29(1.77,10.40) | 0.012 |
| MR-Egger ***intercept*** |  |  | - | 0.037 |
| Q statistic |  |  | - | 1.051e-276 |
| OSA on COPD | | | | |
| IVW | 8 | 441 | 1.00(0.99,1.00) | 0.973 |
| Weighted median |  |  | 1.00(0.99,1.00) | 0.444 |
| MR-Egger |  |  | 1.00(0.99,1.01) | 0.244 |
| MR-Egger ***intercept*** |  |  | - | 0.236 |
| Q statistic |  |  | - | 0.531 |

IVW, inverse variance weighted; SNP, single nucleotide polymorphism; CI, confidence intervals; All statistical tests were two-sided. A P-value< 0.05 was considered significant. BMI,body mass index;COPD, chronic obstructive airways disease.


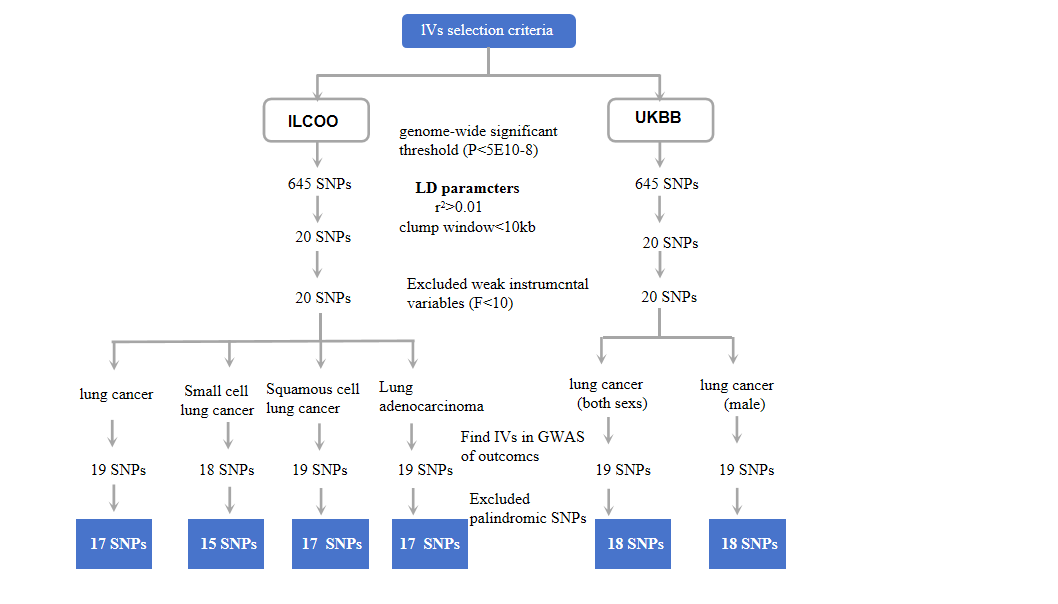


**Figure S1.** Flowchart of the SNP selection process for all univariate MR analyses. IVs instrument variables, MR mendelian randomization, MVMR multivariable mendelian randomization, ILCCO International Lung Cancer Consortium, BMI body mass index, LD linkage disequilibrium, GWAS genome-wide association studies, SNP single nucleotide polymorphism.


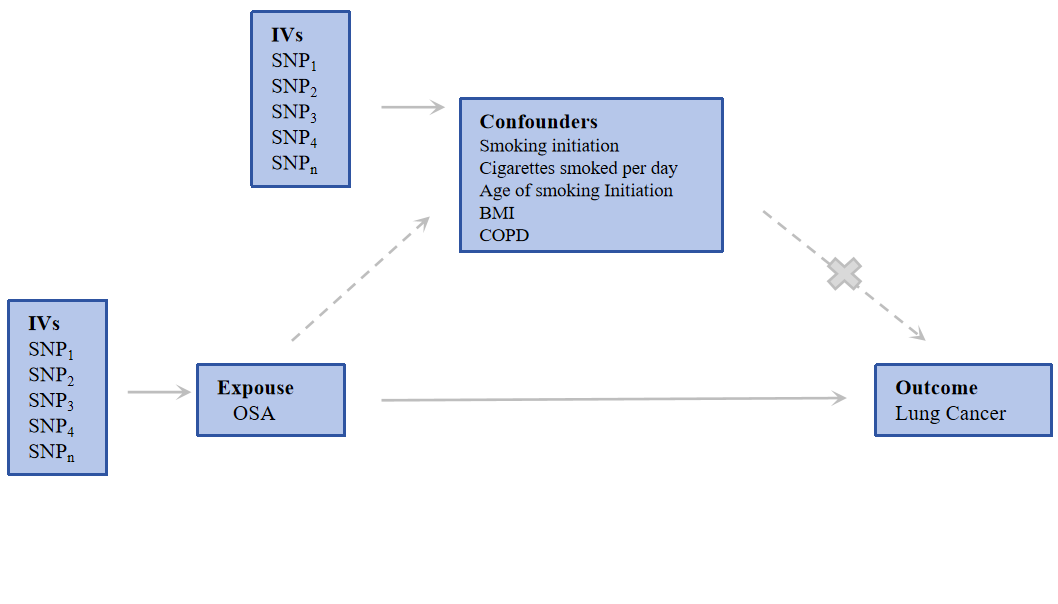


**Figure S2.** The MR association between OSA and lung cancer was not violated through pleiotropic pathways relating to lung cancer. OSA, obstructive sleep apnea; MR mendelian randomization BMI,body mass index;COPD, chronic obstructive airways disease.


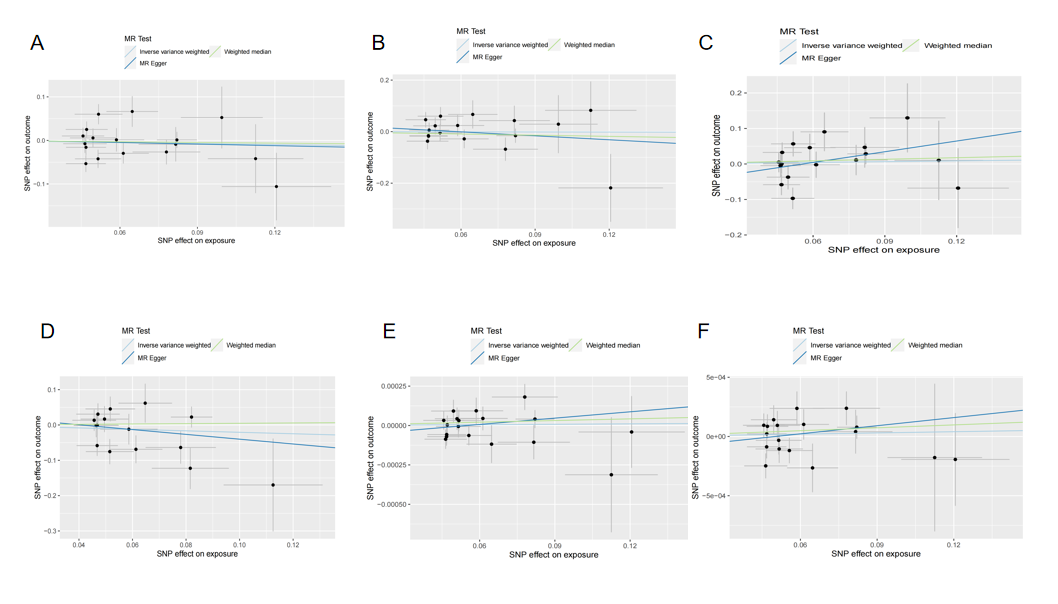


**Figure S3.** Scatter plot of SNPs associated with OSA and their risk of lung cancer. (A) total lung cancer (B) lung adenocarcinoma (C) lung squamous cell carcinoma (D) small cell lung carcinoma (E) total lung cancer(UKB-both sex) (F) total lung cancer(UKB-male)


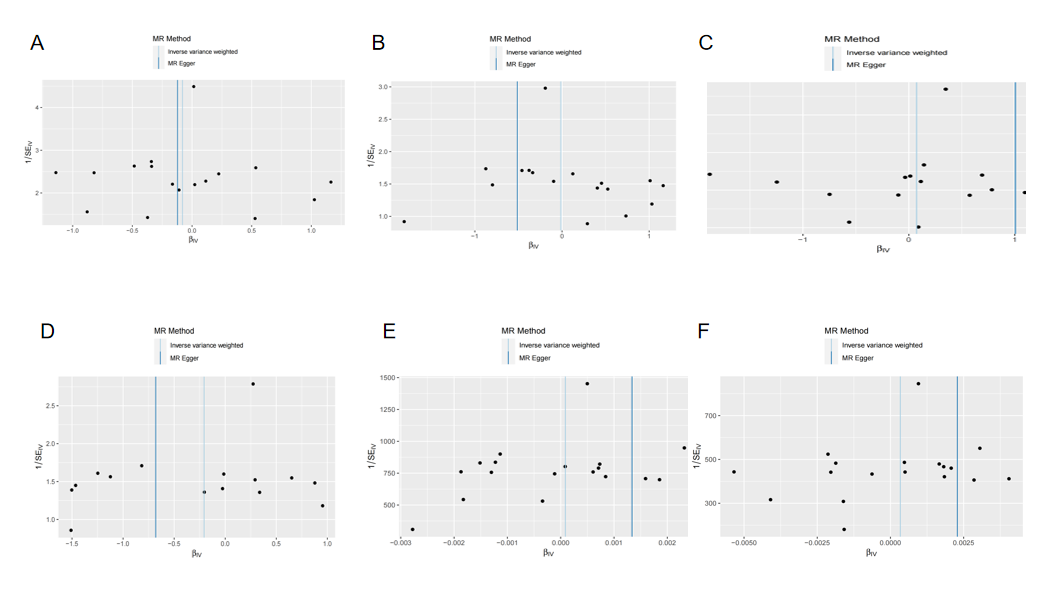


**Figure S4.** Funnel plot of SNPs associated with OSA and their risk of lung cancer. (A) total lung cancer (B) lung adenocarcinoma (C) lung squamous cell carcinoma (D) small cell lung carcinoma (E) total lung cancer(UKB-both sex) (F) total lung cancer(UKB-male)


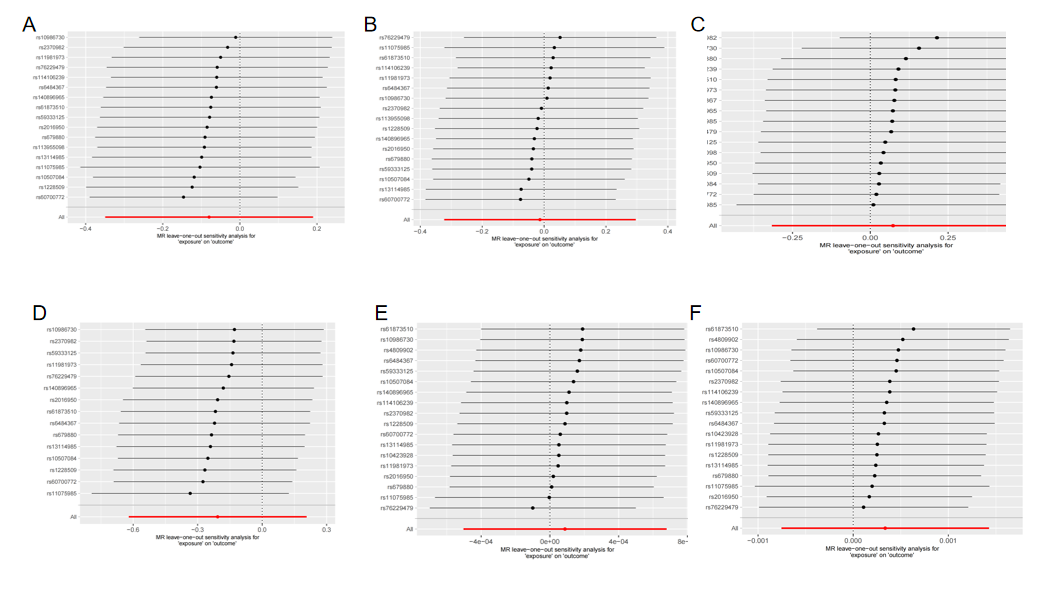


**Figure S5.** Leave-one-out of SNPs associated with OSA and their risk of lung cancer. (A) total lung cancer (B) lung adenocarcinoma (C) lung squamous cell carcinoma (D) small cell lung carcinoma (E) total lung cancer(UKB-both sex) (F) total lung cancer(UKB-male)
